# Laparoscopic-assisted transversus abdominus plane block versus intraperitoneal irrigation of local anesthetic for patients undergoing laparoscopic cholecystectomy: a prospective, multicentre, single-blinded, randomised controlled trial

**DOI:** 10.1007/s00464-026-12649-0

**Published:** 2026-03-10

**Authors:** Matthew G. Davey, David E. Kearney, Sherif El-Masry, Arnold D. K. Hill

**Affiliations:** 1 Department of Surgery, Royal College of Surgeons in Ireland, Dublin 2, Republic of Ireland; 2 Department of Surgery, Connolly Hospital Blanchardstown, Dublin 15, Ireland; 3 Department of Surgery, Our Lady of Lourdes Hospital, Drogheda, Co. Louth Ireland; 4 Department of Surgery, Beaumont Hospital, Dublin 9, Ireland; 5 Our Lady of Lourdes Hospital, Drogheda/Louth County Hospital, Drogheda, Ireland; 6 Connolly Hospital Blanchardstown/Our Lady’s Hospital, Navan, Ireland; 7 Beaumont Hospital/St. Joseph’s Hospital, Raheny, Ireland; 8 Royal College of Surgeons in Ireland, Dublin 2, Republic of Ireland

**Keywords:** Laparoscopic cholecystectomy, Perioperative management, Transversus abdominus plane block, Intraperitoneal irrigation

## Abstract

**Background:**

The PROSPECT guidelines provide GRADE A recommendations for paracetamol, non-steroidal anti-inflammatories, and port site infiltration (PSI) with local anaesthetic following laparoscopic cholecystectomy. Despite varying practice, the optimal method of delivering additional local anesthetic is unclear.

**Aim:**

To perform a randomised clinical trial (RCT) evaluating the value of laparoscopic-delivered transversus abdominal plane block (L-TAP) compared to intraperitoneal infiltration (IP) in addition to PSI in patients undergoing laparoscopic cholecystectomy.

**Methods:**

A multicentre RCT was performed during a 7-month recruitment period (March–October 2025) across 6 hospitals. Patients were randomised on a 1:1 basis to L-TAP or IP. The primary outcome was postoperative visual analogue scores (VAS). Descriptive statistics and regression analyses were performed.

**Results:**

147 patients were recruited, of whom, 135 underwent final analysis. Of these, 49.6% were allocated to IP (67/135) and 50.1% to L-TAP (68/135). A non-significant difference was observed in baseline clinical information between groups (*P* > 0.050). A significant reduction in mean VAS was observed in favour of L-TAP at 6-h (IP: 3.3 (standard deviation (SD): 0.3) vs. L-TAP: 2.3 (SD: 0.3), *P* = 0.014) and 24-h (IP: 3.1 (SD: 0.4) vs. L-TAP: 1.6 (SD: 0.4), *P* = 0.008), with a trend towards significance at 12-h (IP: 3.5 (SD: 0.4) vs. L-TAP: 2.5 (SD: 0.4), *P* = 0.063). Moreover, regression analysis demonstrated a significant reduction in VAS following TAP (beta-coefficient: -0.681, standard error: 0.281, *P* = 0.015), however, a non-significant difference in ‘breakthrough’ opioid and morphine equivalent consumption was noted between groups (*P* > 0.050). There was a non-significant difference in surgical data, postoperative outcomes, and quality of life metrics between groups (*P* > 0.050).

**Conclusion:**

This study demonstrates the superiority of L-TAP compared to IP in reducing postoperative pain, as measured VAS scores, in patients undergoing laparoscopic cholecystectomy.

**Supplementary Information:**

The online version contains supplementary material available at 10.1007/s00464-026-12649-0.

Laparoscopic cholecystectomy remains the cornerstone in treating benign diseases of the gallbladder and biliary tree [1], with approximately 5000 of these procedures being performed annually in the Republic of Ireland [2]. While significant complications, including bile leak, common bile duct injury (CBDI), and converting to an open incision, are typically discussed in detail when counselling patients in the preoperative setting [3], post-operative pain in the first 24-h after surgery is typically less well detailed and remains the main barrier of early discharge following this procedure [4]. To counteract this issue, multimodal analgesic strategies have been adopted with the objective of minimising post-operative pain following laparoscopic cholecystectomy, as outlined by the Procedure Specific Post-Operative Pain Management (PROSPECT) in their recent review and recommendations [5]. More specifically, the PROSPECT guidelines provide GRADE A recommendations in support of both standard paracetamol and non-steroidal anti-inflammatory (or cyclooxygenase-2-specific inhibitors), combined with surgical site local anaesthetic infiltration in patients in the peri-operative setting ‘as the first line for routine use’ [5]. This combination represents the standard practice at present in the Republic of Ireland.

Despite these recommendations, there remains ambiguity in the surgical literature surrounding to the optimal strategy for the infiltration of local anaesthetic in tandem with port-site infiltration (PSI); previous data have coherently demonstrated the superiority of surgical site wound infiltration, intraperitoneal infiltration and intra-abdominal wall blocks with local anaesthetic agents in reducing post-operative pain relative to placebo [4, 6, 7]. In particular, data from a previous randomised controlled trial (RCT) has indicated that intraperitoneal infiltration (IP) of the gallbladder fossa with local anaesthetic is more effective in reducing post-operative pain than PSI [8], however, these results were subsequently challenged and refuted in a sequential RCT [9], leaving ambiguity as to the effect of this technique in reducing post-operative pain. Furthermore, recent RCTs have demonstrated that infiltration of local anaesthetic into the transversus abdominus plane (TAP) or the rectus sheath intraoperatively have successfully reduced post-operative pain compared to PSI alone, which has been translated directly into a reduction in post-operative morphine requirement [10, 11]. While ultrasound-guided transversus abdominus plane (US-TAP) was often deployed to optimise pain in the perioperative setting following laparoscopic cholecystectomy [12], the suitability of a laparoscopic-guided approach (L-TAP) has recently been ratified as non-inferior to the ultrasound-guided approach in a recent meta-analysis of prospective RCTs, which was performed by the Principal Investigators of the current study [13].

Given the contrasting results of these previously reported studies, there remains no consensus as to the validity of using L-TAP or IP of local anaesthetic to the liver bed as contemporary modes of delivering local anaesthetic following laparoscopic cholecystectomy.

Accordingly, the aim of this study was to perform a prospective, RCT evaluating the value of adding L-TAP or IP to standard PSI in patients indicated to undergo laparoscopic cholecystectomy. This study is a parallel two-arm trial which evaluated outcomes following local anesthetic infiltration [1] directly into the laparoscopic port sites combined with IP, and [2] directly into the laparoscopic port sites combined with laparoscopic-delivered TAP block.

## Methods

### Study design

This study was a prospective, multicentre, single-blinded randomised controlled trial designed in accordance with the CONSORT guidelines for prospective, parallel group randomised studies [14]. This study was a standard two-group parallel designed study which recruited patients from six sites in the RCSI Hospital network (i.e.: Beaumont Hospital, St. Josephs’ Hospital Raheny, Our Lady of Lourdes Hospital, Drogheda, Louth County Hospital, Connolly Hospital Blanchardstown, and Our Lady’s Hospital Navan) in the North East of the Republic of Ireland. This study was prospectively registered prior to study commencement (NCT06714279).

Patients were considered for recruitment into this study if they are indicated to undergo elective laparoscopic cholecystectomy. Thereafter, they were randomised into two respective treatment arms; [1] PSI combined with IP infiltration, and [2] PSI combined with L-TAP.

### Main study summary

This study was conducted over a period of 7-months (March 2025 – October 2025), where adult patients aged 18 years or older attending any of the recruitment sites for laparoscopic cholecystectomy were considered for inclusion. Randomisation was generated on a 1-to-1 basis via a secure web service, Sealed Envelope (Sealed Envelope Ltd., London, United Kingdom). The random allocation was to [1] local anesthetic delivery directly PSI combined with IP, and [2] local anesthetic delivery with PSI combined with L-TAP.

Participating patients all had clinical follow–up for 4–6 weeks post-operatively, in accordance with the preference of the attending surgeon. Visual Analogue Scale (VAS) measurements were collected at 1-h, 3-h, 6-h, 12-h and 24-h post-procedure. Further opioid analgesic requirements will also be recorded for during inpatient stay and on discharge from hospital. The total opioid consumption by each patient was measured using morphine equivalents. Patient monitoring and follow-up was performed by the clinical teams responsible for the management of these patients post-operatively, with an additional follow-up phone call from the study collaborators made to determine the impact of surgery on patient quality of life (QOL) and final VAS.

### Treatment arms

**Fig. 1:**
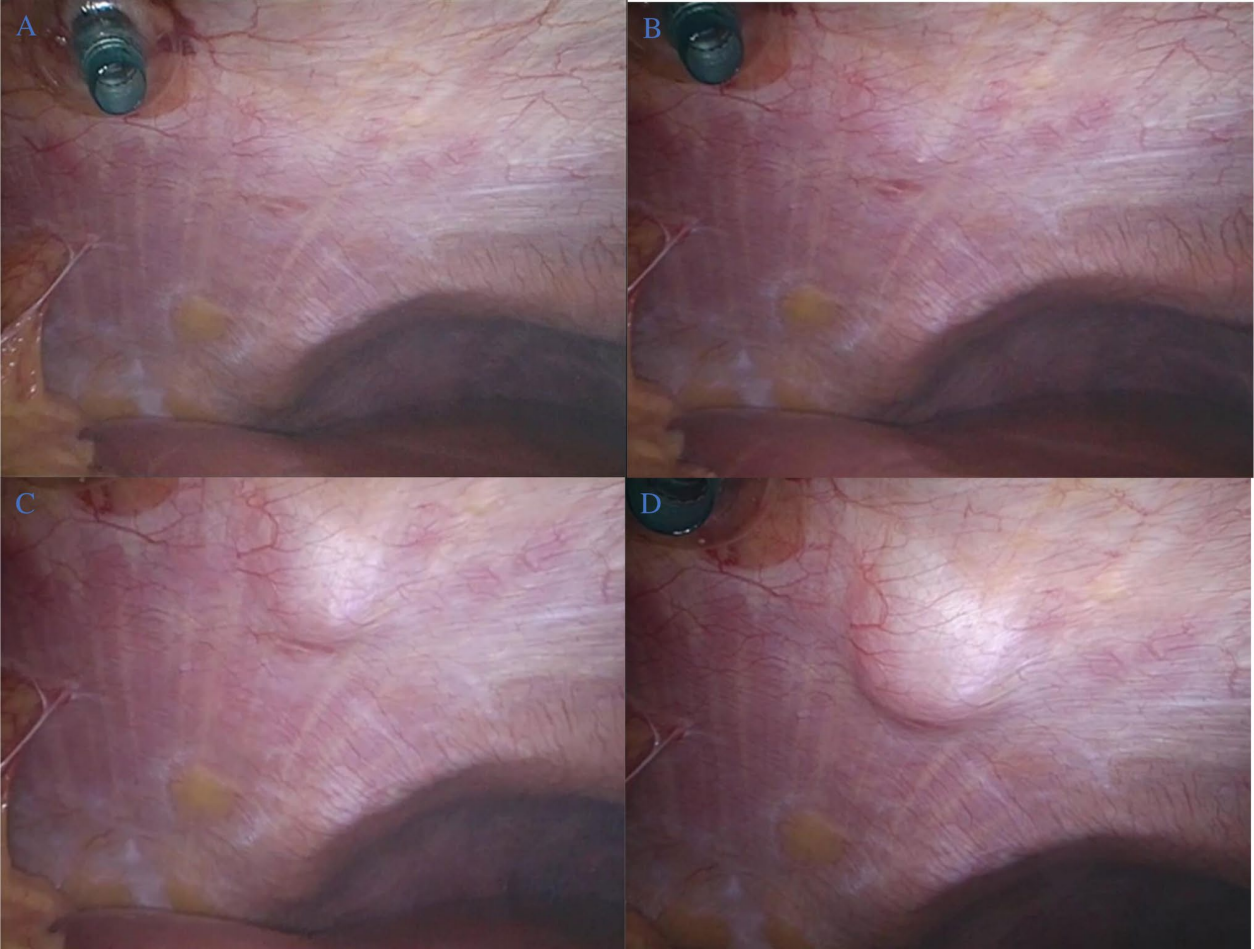
Scheme outlining the step-by-step laparoscopic-delivered transversus abdominus plane block delivery; **A** direct visualisation from inside the abdominal cavity during laparoscopy, **B** advancing the local anesthetic needle tip into the abdominal wall toward the preperitoneal space (circled in green), **C** confirmation of the plane being ensured by direct visualisation of a uniform protrusion downwards of the transversus abdominus muscle fibres, which is known as ‘Doyle’s bulge’, and **D** observing a direct preperitoneal ‘bulge’ laparoscopically indicates that the infiltration is deeper to the correct plane, indicating that the needle should be withdrawn more superficially prior to continuing infiltration

Preparation of Levobupivacaine 2.5 mg/mL was made up to the maximum dose of 2 mg/kg (or a maximum dose of 150 mg, in accordance with the Health Products Regulatory Authority instructions).

(A) Local anesthetic infiltration into the laparoscopic port sites combined with IP; this treatment arm involved direct infiltration of Levobupivacaine onto the liver bed after completion of the procedure and administration into laparoscopic port-site wounds immediately prior to wound closure. A 50:50 split in local anesthetic administration was distributed to intraperitoneal infiltration and the port-site infiltration, respectively.

(B) Laparoscopic-guided infiltration of local anesthetic via TAP block with combined laparoscopic PSI; this treatment arm involved direct infiltration of Levobupivacaine into the preperitoneal space above the rectus sheath under direct vision during laparoscopy. This block involved the direct visualisation from inside the abdominal cavity during laparoscopy, just before the removal of laparoscopic ports and commencing the deflation the abdomen. The operating surgeon then advanced the local anesthetic needle tip into the abdominal wall toward the preperitoneal space. Once the needle tip was visualised in the peritoneum at laparoscopy, the needle was slowly withdrawn to a level approximately 0.5 cm superficial to the transversus abdominus muscle. The surgeon then infiltrated the local anesthetic into this plane (i.e.: the transversus abdominus plane). Confirmation of the plane was then ensured by direct visualisation of a uniform protrusion downwards of the transversus abdominus muscle fibres, which is known as ‘*Doyle’s bulge*’. Seeing a direct preperitoneal or muscle ‘blister’ or ‘bulge’ laparoscopically indicated that the infiltration is deeper to the correct plane, indicating that the needle should be withdrawn more superficially prior to continuing infiltration. A 50:50 split in local anesthetic administration was evenly allocated to TAP block and the port-sites, respectively. Figure 1 provides a step-by-step schema of L-TAP being delivered.

### Outcome measures

#### Primary outcome measures

The primary outcome measures for the study was to quantify and analyse differences in the post-operative pain as measured using VAS at 1-h, 3-h, 6-h, 12-h and 24-h following laparoscopic cholecystectomy. Thereafter, the further opioid analgesic requirements for each patient was recorded for the duration of their inpatient stay and also the total sum of opioids prescribed for these patients on discharge (measured using morphine equivalents).

#### Secondary outcome measures

The secondary outcome measures for the study were to obtain information regarding time to ambulation (measured in hours), time to discharge (measured in hours), early (i.e.: atelectasis, lower respiratory tract infections, conversion to open, etc.) and late (i.e.: bile leak, retained common bile duct stone, common bile duct injury (CBDI), etc.) complications, and additional interventions, operations and readmissions related to their index procedure were recorded.

#### Power calculation

Based on statistical input from our colleagues in the Department of Mathematics and Statistics, allowing for a mean VAS of 3.5 with standard deviation of 1.5, an estimated 25% reduction would determine that 126 patients were required to achieve 90% power at the 5% significance level, (63 in each arm). To allow for losses to follow-up and withdrawals, 10% was added to each arm to ensure adequate power was achieved. Therefore, the total projected sample size for this study was 140 patients.

#### Ethical approval

The study was designed in accordance with principles of the Declaration of Helsinki and “good clinical practice” guidelines [15]. This study was granted local hospital ethical approval by the local hospital clinical research ethics committees at each site prior to the commencement of trial recruitment [Beaumont Hospital / St. Josephs’ Hospital Raheny (REC/24/72), Our Lady of Lourdes Hospital, Drogheda / Louth County Hospital (REC/25/002), Connolly Hospital Blanchardstown / Our Lady’s Hospital Navan (REC/CHB/001/25)]. Written informed consent was prospectively obtained preoperatively. This study received no financial aid.

#### Randomisation

Randomisation was performed on a one-to-one basis using a computer randomisation programme with the minimisation algorithm to ensure a balanced allocation of patients across the two treatment groups. Participants were randomised using simple randomisation in order to seed the minimisation algorithm which will have a probable cystic element incorporated to ensure unpredictability of treatment assignment. Randomisation group was confirmed by the treating surgeon at the beginning of the operative procedure. Participants were blinded to their intervention.

#### Statistical analysis

Standard statistical summaries and graphical plots were presented for the primary outcome measure and all secondary outcome measures. Baseline data were summarised to check comparability between treatment arms.

The main analysis investigated the differences in the primary outcome measure, the differences in the post-operative pain, as measured using VAS at 1-h, 3-h, 6-h, 12-h and 24-h following laparoscopic cholecystectomy. A mixed-effects linear regression model was adopted in this study to assess the effects of the interventions on VAS that, for the purposes of analysis, were assumed to be approximately normally distributed. For example, this model was used to assess differences in VAS scores between the study intervention groups, with results presented as beta-coefficient and standard error (SE), with associated 95% confidence intervals (CIs). Other dichotomous outcome variables, such as complications related to the study interventions, were analysed using the conventional linear (fixed-effects) regression approach to a mixed-effects logistic regression analysis.

The primary focus of this study was to compare the two treatment groups on an intention-to-treat (ITT) basis, and this will be reflected in the analysis which will be reported together with appropriate diagnostic plots that check the underlying model assumptions. All reported tests were two-sided and considered to provide evidence for a significant difference if p-values were less than 0.05 (5% significance level). Data were analysed using Statistical Package for Social Sciences™ (SPSS™) version 26.0 (International Business Machines Corporation, Armonk, NY).

## Results

### Participants

**Fig. 2:**
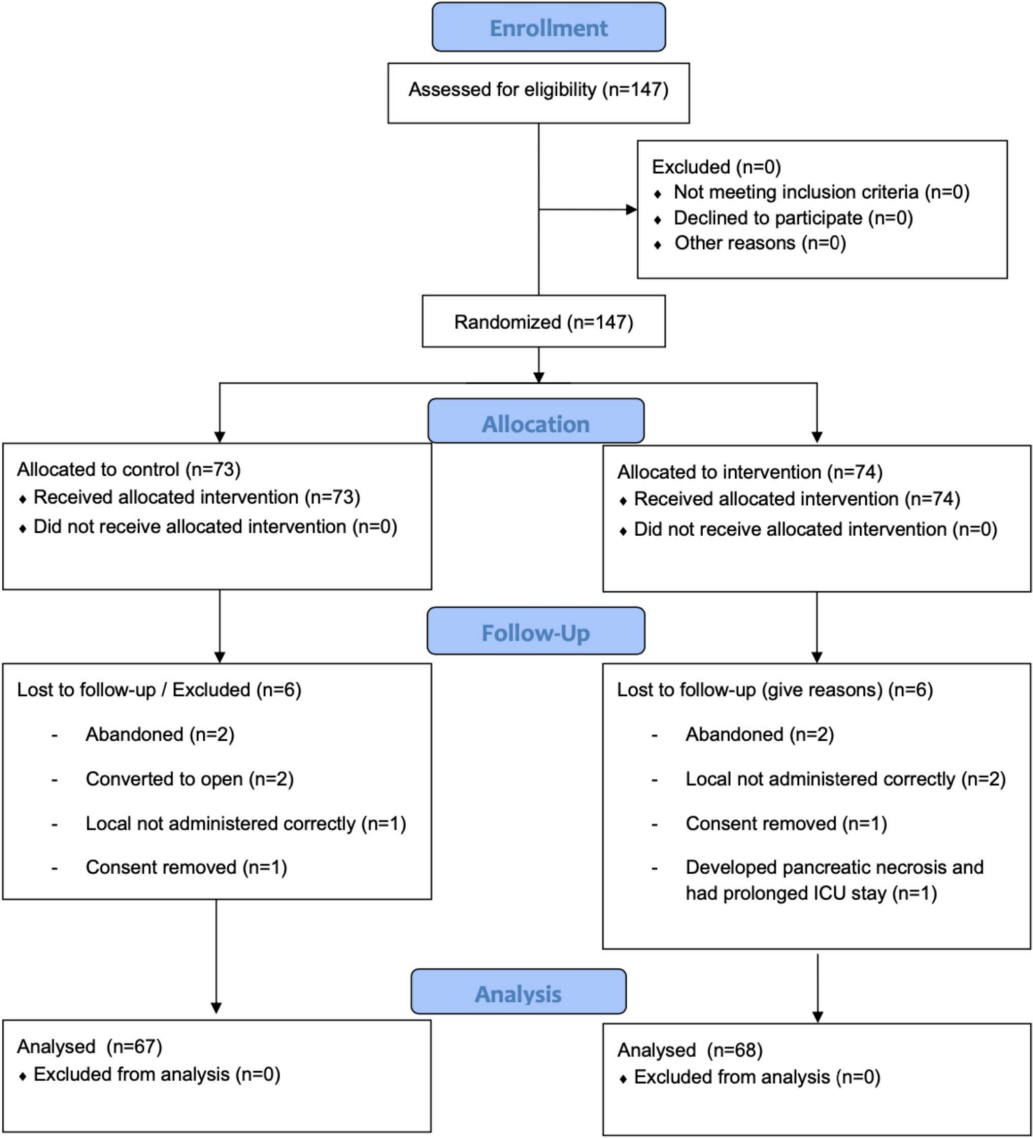
CONSORT diagram demonstrating the trial enrolment, randomisation, and allocation process

During the trial recruitment period (March 2025 – October 2025), 147 patients were initially recruited to this study, of whom, 135 were included for final analysis. In total, 49.6% of patients were allocated to the IP (control) (67/135) and 50.1% to the L-TAP (intervention) arms, respectively (68/135). Of total included patients, 51.1% were recruited from Our Lady of Lourdes Hospital, Drogheda / Louth County Hospital (69/135), while 24.4% were recruited from the Beaumont Hospital / St. Josephs’ Hospital, Raheny (33/135) and Connolly Hospital Blanchardstown / Our Lady’s Hospital Navan (33/135) sites, respectively. There was a non-significant difference in baseline clinical data between each intervention group (Table 1) and specific details regarding patient enrolment, allocation, follow-up, and analysis are outlined in detail in the CONSORT flow chart (Fig. 2).

**Table 1:** Characteristics by intervention group

	IP (*n* = 67, 49.6%)	TAP (*n* = 68, 50.4%)	*P* = Value
Age	49.4 (SD: 1.7) (*n* = 67)	52.0 (SD: 2.0) (*n* = 68)	0.333†
Age category			
< 40	18 (26.9%)	15 (22.1%)	0.381‡
40–65	38 (56.7%)	35 (51.5%)	
> 65	11 (16.4%)	18 (26.5%)	
BMI	30.2 (SD: 0.7) (*n* = 67)	28.7 (SD: 0.7) (*n* = 67)	0.100†
BMI category			
Normal < 25	9 (13.4%)	16 (23.9%)	0.234‡
Overweight 25–30	25 (37.3%)	25 (37.3%)	
Obese > 30	33 (49.3%)	26 (38.8%)	
Smoker			
No	48 (71.6%)	55 (80.9%)	0.510‡
Ex-smoker	9 (13.4%)	6 (8.8%)	
Smoker	10 (14.9%)	7 (10.3%)	
Diabetic			
No	59 (88.1%)	64 (94.1%)	0.243‡
Yes	8 (11.9%)	4 (5.9%)	

*IP* intraperitoneal, *TAP* transversus abdominus plane block, *SD* standard deviations, *n* number, *BMI* body mass indices

† denotes pooled t-test

‡ denotes Fisher’s Exact test

### Surgical characteristics

There was a non-significant difference in the proportion of ‘hot’ gallbladders performed during this study, with a similar intraoperative duration and EBL for patients undergoing both interventions. Surgical data between each intervention group is outlined in detail Table 2.

**Table 2:** Surgical characteristics by intervention group

	IP 67 (49.6%)	TAP 68 (50.4%)	Test
Hot or Cold			
Cold	63 (94.0%)	61 (89.7%)	0.531‡
Hot	4 (6.0%)	7 (10.3%)	
Duration (minutes)	83.1 (SD: 3.3) (*n* = 66)	79.4 (SD: 3.0) (*n* = 67)	0.416†
EBL (millilitres)	31.3 (SD: 5.3) (*n* = 63)	52.5 (SD: 14.0) (*n* = 65)	0.167†
Drain			
No	62 (92.5%)	59 (86.8%)	0.398‡
Yes	5 (7.5%)	9 (13.2%)	

*IP* intraperitoneal, *TAP* transversus abdominus plane block, *SD* standard deviations, *n* number, *EBL* estimated blood loss

† denotes pooled t-test

‡ denotes Fisher’s Exact test

### Postoperative pain outcomes

A significant reduction in postoperative VAS scores in favour of L-TAP compared to IP was observed at 6-h (*P* = 0.014) and 24-h (*P* = 0.008), respectively, with a trend towards statistical significance noted at 12-h (*P* = 0.063) (Table 3). Regression analysis demonstrated a significant reduction in postoperative VAS score for those undergoing L-TAP relative to IP (beta-coefficient: -0.681, SE: 0.281, 95% confidence intervals: -1.231—-0.131, *P* = 0.015). Individual patient VAS scores for those undergoing IP and L-TAP are outlined in Supplementary Material 1 and 2. There was a non-significant difference in ‘breakthrough’ opioid consumption and morphine equivalent consumption between both groups (Table 3).

**Table 3:** Postoperative pain outcomes by intervention group

	IP (*n* = 67, 49.6%)	TAP (*n* = 68, 50.4%)	*P*-Value
VAS 1 h	3.0 (SD: 0.3) (*n* = 66)	2.5 (SD: 0.3) (*n* = 68)	0.197†
VAS 3 h	2.6 (SD: 0.3) (*n* = 66)	2.3 (SD: 0.3) (*n* = 68)	0.452†
VAS 6 h	3.3 (SD: 0.3) (*n* = 60)	2.3 (SD: 0.3) (*n* = 63)	0.014†*
VAS 12 h	3.5 (SD: 0.4) (*n* = 43)	2.5 (SD: 0.4) (*n* = 41)	0.063†
VAS 24 h	3.1 (SD: 0.4) (*n* = 35)	1.6 (SD: 0.4) (*n* = 32)	0.008†*
Breakthrough opioids			
No	19 (28.4%)	21 (30.9%)	0.851‡
Yes	48 (71.6%)	47 (69.1%)	
Morphine equivalents (mg)	16.9 (SD: 2.3) (*n* = 67)	13.6 (SD: 1.6) (*n* = 68)	0.245†

*IP* intraperitoneal, *TAP* transversus abdominus plane block *SD*, standard deviations, *n* number, *VAS* visual analogue score

† denotes pooled t-test

‡ denotes Fisher’s Exact test

*denotes statistical significance

### Postoperative outcomes

A non-significant difference in postoperative time to ambulation, frequency of ambulation, metres mobilised, time to flatus, length of hospital stay, and postoperative nausea and vomiting between both groups (Table 4). A non-significant difference in post-operative complications was observed (*P* = 0.791). A detailed breakdown in postoperative complications are outlined in Table 4.

**Table 4:** Postoperative outcomes by intervention group

	IP (*n* = 67,49.6%)	TAP (*n* = 68,50.4%)	*P*-Value
Time to ambulation (hours)	5.6 (SD,0.5) (*n* = 66)	5.2 (SD,0.4) (*n* = 63)	0.481†
Frequency ambulation	2.6 (SD,0.3) (*n* = 58)	2.6 (SD,0.2) (*n* = 56)	0.971‡
Metres mobilised	49.0 (SD,15.0) (*n* = 55)	31.5 (SD,4.6) (*n* = 51)	0.282†
Time to flatus (hours)	10.3 (SD,1.8) (*n* = 30)	9.6 (SD,1.4) (*n* = 25)	0.766†
LOS (hours)	28.8 (SD,5.3) (*n* = 67)	20.1 (SD,1.9) (*n* = 68)	0.123†
POV/retching			
None	55 (82.1%)	54 (79.4%)	0.603‡
Once	11 (16.4%)	12 (17.6%)	
Twice	1 (1.5%)	0 (0.0%)	
Three or more	0 (0.0%)	2 (2.9%)	
PONV ADLs			
Not at all	58 (86.6%)	58 (85.3%)	0.723‡
Sometimes	8 (11.9%)	7 (10.3%)	
Often	1 (1.5%)	3 (4.4%)	
Post-operative laxatives			
No	62 (92.5%)	65 (98.5%)	0.208‡
Yes	5 (7.5%)	1 (1.5%)	
Complications			
No	60 (89.6%)	59 (86.8%)	0.791‡
Yes	7 (10.4%)	9 (13.2%)	
LRTI			
No	66 (98.5%)	68 (100.0%)	0.496‡
Yes	1 (1.5%)	0 (0.0%)	
VTE			
No	66 (98.5%)	67 (98.5%)	1.000‡
Yes	1 (1.5%)	1 (1.5%)	
SSI			
No	66 (98.5%)	66 (97.1%)	1.000‡
Yes	1 (1.5%)	2 (2.9%)	
Intra-abdominal collection			
No	66 (98.5%)	68 (100.0%)	0.496‡
Yes	1 (1.5%)	0 (0.0%)	
Post-operative bleed (monitoring)			
No	65 (97.0%)	63 (92.6%)	0.441‡
Yes	2 (3.0%)	5 (7.4%)	
Post-operative bleed (transfusion)			
No	67 (100.0%)	67 (98.5%)	1.000‡
Yes	0 (0.0%)	1 (1.5%)	
Retained CBD stone			
No	66 (98.5%)	68 (100.0%)	0.496‡
Yes	1 (1.5%)	0 (0.0%)	
Bile leak			
No	66 (98.5%)	68 (100.0%)	0.496‡
Yes	1 (1.5%)	0 (0.0%)	
Reintervention			
No	64 (95.5%)	68 (100.0%)	0.119‡
Yes	3 (4.5%)	0 (0.0%)	
Reoperation			
No	66 (98.5%)	68 (100.0%)	0.496‡
Yes	1 (1.5%)	0 (0.0%)	
Readmission within 30 days			
No	65 (97.0%)	68 (100.0%)	0.244‡
Yes	2 (3.0%)	0 (0.0%)	

*IP* intraperitoneal, *TAP* transversus abdominus plane block, *SD* standard deviation, *n* number, *LOS* length of stay, *POV* postoperative vomiting, *POVN* postoperative vomiting or nausea, *ADL* activities if daily living, *LRTI* lower respiratory tract infection, *VTE* venothromboembolic disease, *SSI* surgical site infection, *CBD* common bile duct

† denotes pooled t-test

‡ denotes Fisher’s Exact test

Patient satisfaction, quality of life outcomes and final VAS scores are outlined in detail in Table 5. The final histology reports from included patients are outlined in brief in Supplementary Material 3.

**Table 5:** Quality of life outcomes and final pain scores by intervention group

	IP (*n* = 67,49.6%)	TAP (*n* = 68,50.4%)	P-Value
Mean EQ-5D-5L index	0.9 (SD,0.0) (*n* = 64)	0.9 (SD,0.0) (*n* = 64)	0.171†
Patient Satisfaction	65/67 (97.0%)	68/68 (100.0%)	0.244‡
Final VAS			
0	34 (64.2%)	42 (77.8%)	0.545‡
1	6 (11.3%)	2 (3.7%)	
2	6 (11.3%)	5 (9.3%)	
3	4 (7.5%)	4 (7.4%)	
4	2 (3.8%)	1 (1.9%)	
5	1 (1.9%)	0 (0.0%)	

*IP* intraperitoneal, *TAP* transversus abdominus plane block, *SD* standard deviations, *n* number, *VAS *visual analogue score

† denotes pooled *t*-test

‡ denotes Fisher’s Exact test

## Discussion

This prospective, RCT was designed with the ambition of recruiting prospective patients who were indicated to undergo laparoscopic cholecystectomy across 6 recruitment centres in the RCSI Hospital Network and randomising them to either laparoscopically-delivered TAP block compared to intraperitoneal irrigation of local anaesthesia over the liver, in a blinded manner. To the authors knowledge, this is the first prospective, multicentre RCT which was designed to compare the efficacy of these local anaesthetic administration strategies (which are solely deliverable by the operating surgeon) in patients undergoing laparoscopic cholecystectomy. The most important findings in this trial was the data in support of TAP block deployment as a means of reducing postoperative pain, as measured using VAS scores, which indicate robust L-TAP block administration should be considered in contemporary surgical practice, in particular, given the common propensity for surgeons to perform intraperitoneal delivery of local anaesthesia as routine to optimise postoperative pain following laparoscopic cholecystectomy. These results are novel in that both methods of local anaesthesia administration may be performed with relative ease by the operating surgeon, mitigating the requirement for the involvement of other specialty services in attempt to optimise postoperative pain in patients undergoing this procedure.

With respect to the primary outcome measures of interest, L- TAP block was associated with an overall reduction in mean VAS scores at 1-, 3-, 6-, 12-, and 24-h, including a significant difference observed at 6-h (*P* = 0.014) and 24-h (*P* = 0.008), respectively, and a trend towards significance at 12-h (*P* = 0.014). These are important findings, which may be fruitful in enhancing patient experience in the postoperative setting, in particular, with the knowledge that postoperative pain remains among the most common barriers to early discharge following laparoscopic cholecystectomy, as outlined in detail by Xiong et al. in their review [16]. Notwithstanding these important results, it is of course plausible that one may deduce a theory suggesting that these results may have been perhaps somewhat predictable: TAP blockade involves the direct infiltration of local anaesthetic agents into the transversus abdominal plane, wherein, the anterior primary rami of the T7-T12 spinal nerves pass deep to the internal oblique muscle prior to perforating through the rectus abdominus and providing cutaneous segmental cutaneous innervation to the anterior abdominal wall [17]. As is standard practice, this is where the typical incisions for laparoscopic cholecystectomy are routinely performed [18]. Conversely, intraperitoneal irrigation of local anaesthetic to the liver bed following cholecystectomy seemingly provides a less targeted approach, whereby external factors, such as changes to patient positioning, the smooth contour and shape of the liver parenchyma, and the force gravity, all likely negatively impact the drug absorption and efficacy. Moreover, there is data indicating that local anaesthetic infiltration into targeted spaces increases absorption and peak plasma concentration [19]. Therefore, an a priori deduction may have been preconceived in support of L-TAP, which has subsequently been supported by the data presented in the results of this RCT. Therefore, this study indicates that L-TAP serves as an effective means of reducing postoperative pain following laparoscopic cholecystectomy and should be considered by those performing this procedure.

Interestingly, TAP was initially pioneered by our colleagues in the field of anaesthesia [20, 21]. The Principal Investigators of this study have previously published a meta-analysis of 428 patients from six prospective RCTs which demonstrated the clinical equipoise of L-TAP and US-TAP in reducing postoperative pain following laparoscopic cholecystectomy, with a significant reduction in the time taken to perform L-TAP observed [13]. These results, taken in tandem with the results of the present RCT, indicate the pragmatism associated with L-TAP use for this purpose. Thus, the authors of this study encourage surgeons to perform L-TAP as routine for their prospective patients undergoing laparoscopic cholecystectomy as a safe means of optimising postoperative pain.

In this RCT, L-TAP significantly reduced postoperative VAS following laparoscopic cholecystectomy, while also leading to an overall reduction in modifications in opioid consumption, morphine equivalent consumption, or length of hospital stay. Notwithstanding these positive outcomes, these results did not translate to a significant change in the aforementioned secondary outcomes of interest. It is important to acknowledge that the power calculation performed for this study was conducted based on a proposed significant difference in the primary outcome measure (i.e.: VAS post-operatively), which has been successfully demonstrated at both 6- and 24-h follow-up. Accordingly, given this trial design, it is unlikely that these data are adequately powered to decipher clinically relevant differences between these secondary outcome measures of interest (i.e.: morphine equivalent and ‘breakthrough’ opioid consumption). Notwithstanding this, we do note that in this multicentre RCT, the incidence of post-operative surgical complications were directly comparable with previously published studies comprised ‘real world’ data (i.e.: one bile leak which was managed conservatively (0.7%), one patient who bled postoperatively requiring transfusion (0.7%), one patient who developed a retained CBD stone (0.7%) and no CBDI (0.0%)) [22]. In addition, readmission rates following surgery for IP and L-TAP were 3.0% and 0.0%, respectively, which are well within the accepted readmission rates in the literature, as outlined by McIntyre et al. in their systematic review of over 1.5 million cholecystectomies [23].

In conclusion, this prospective, multicentre, single-blinded, RCT demonstrated the superiority of L-TAP compared to IP in reducing postoperative pain, as measured VAS scores, in patients undergoing laparoscopic cholecystectomy. In essence, the Principal Investigators of this study advocate for surgeons to perform L-TAP as routine for their prospective patients undergoing laparoscopic cholecystectomy, given its value in mitigating postoperative pain, as illustrated in this study.

## Supplementary Information

Below is the link to the electronic supplementary material.

Supplementary file1 (DOCX 220 KB)

Supplementary file 2 (DOCX 15 KB)
