# Supplementary material for: Laparoscopic-assisted transversus abdominus plane block versus intraperitoneal irrigation of local anesthetic for patients undergoing laparoscopic cholecystectomy: a prospective, multicentre, single-blinded, randomised controlled trial
Source: Surg Endosc. 2026 Mar 10;40(5):4263–73. doi: 10.1007/s00464-026-12649-0 (PMC13160988; doi:10.1007/s00464-026-12649-0)
Supplement: Supplementary file 1 — Supplementary file1 (DOCX 220 KB) [file 464_2026_12649_MOESM1_ESM.docx]

**Supplementary Material 1.** Individual patient visual analogue scores for those undergoing intraperitoneal infiltration with local anesthetic.

**
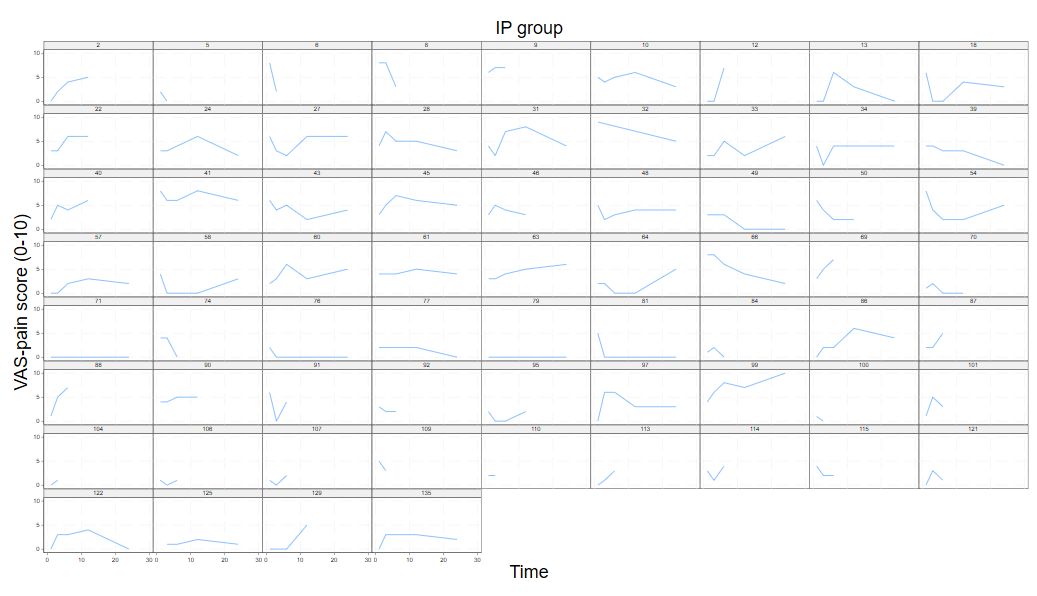
**

**Supplementary Material 2.** Individual patient visual analogue scores for those undergoing laparoscopic transversus abdominal plane block.

**
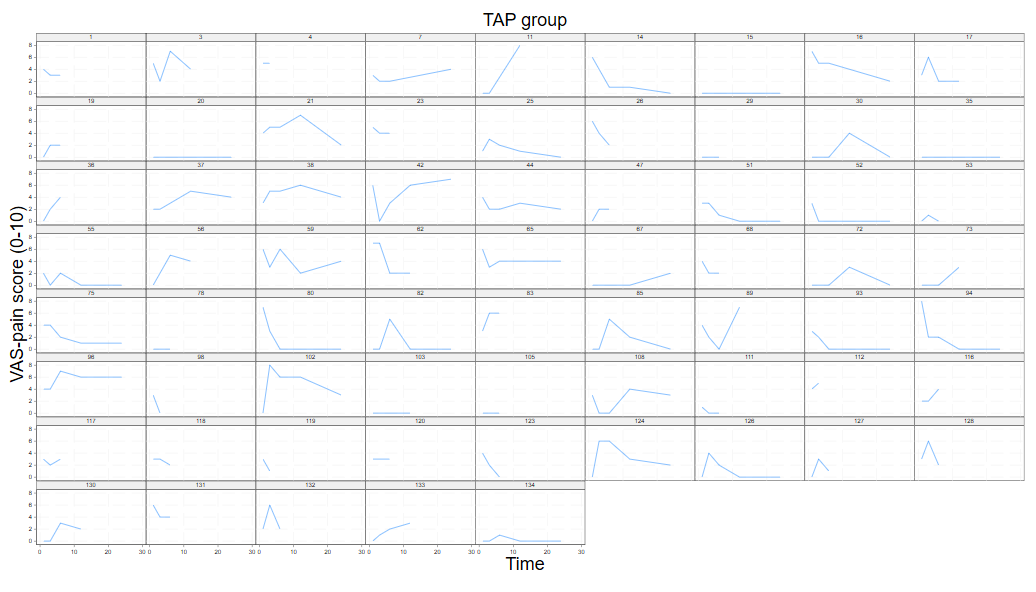
**

**Supplementary Material 3.** Final histology reports for patients included in this study.

| Final Histology | Number of patients (%) |
| --- | --- |
| Chronic Cholecystitis | 110 (81.5%) |
| Acute/acute-on-chronic Cholecystitis | 12 (8.9%) |
| Intestinal metaplasia with low grade dysplasia | 3 (2.2%) |
| Low grade biliary intraepithelial neoplasia, wild type p53 expression | 1 (0.7%) |
| Unavailable | 9 (6.7%) |
