# Supplementary material for: Laparoscopic-assisted transversus abdominus plane block versus intraperitoneal irrigation of local anesthetic for patients undergoing laparoscopic cholecystectomy: a prospective, multicentre, single-blinded, randomised controlled trial
Source: Surg Endosc. 2026 Mar 10;40(5):4263–73. doi: 10.1007/s00464-026-12649-0 (PMC13160988; doi:10.1007/s00464-026-12649-0)
Supplement: Supplementary file 2 — (DOCX 15 KB) [file 464_2026_12649_MOESM2_ESM.docx]

**Principal Investigators:**

Matthew G Davey, David E. Kearney, Sherif El-Masry, Arnold D.K. Hill

**TAP CHOLE Trial Collaboration Authorship list:**

**Our Lady of Lourdes Hospital, Drogheda/Louth County Hospital**

Supyae Yadanar, Emma Forde, Claire Keohane, Hussein Suliman, Arvin Perthani, Aisha Osman Alseikh Alzain, Mohammed Elfaki, Maheen Rana, Emilie McCormack, Yasmine Roden, Wael Shabo, Mohammed Amir, Ammar Ahmed, Mohammed Salama, Babur Sami, Aine O’Neill, Waqar, Aziz Hamad, Ayah Musa, Brendan Moran, Oliver Barrett, Abdel Nasr, Chaudhri Shahbaz, Eleanor Faul, Masaoud Bashir, Obai Elzamzami, Haresh Kumar, Ibrahim Ahmed

**Connolly Hospital Blanchardstown/Our Lady’s Hospital, Navan**

Lucy Burns, Muhammad Zeshan, Caroline Drumm, Niamh Smyth, Ciara Hunt, Kaotharat Balogun, Eoghan Kennedy, Mohammed Alazzawi, Sumaira Zulfiqar, Naveed Abbas, Vikram Tewaitia, Himanshu Yadav, James Byrne, Paula Loughlin, Michael J, Allen, Mayilone Arumugasamy, Achille Mastrosimone, David Beddy

**Beaumont Hospital/St. Joseph’s Hospital, Raheny**

Amira Amir, Yumna Asif, Nicola McShane, Domhnall O’Connor, Fiachra McHugh, Muhammad Assam Sarwar, Gordan Daly, Jake McDonnell, Noel E. Donlon, Eanna J Ryan, Colm Neary, Angus Lloyd, Jennifer McGarry, William Duggan, John P Burke, Niamh McCawley, Mohammed Aafik, Brenda Murphy, Colm Power, Jarlath C. Bolger, William B Robb, Ian S Reynolds, Abeeda Butt, Darren Porter, Trudi Roche, Sorcha O’Grady

**Royal College of Surgeons in Ireland**

Jan Sorensen
